# Pain, quality of life and activity in aged evacuees living in temporary housing after the Great East Japan earthquake of 11 March 2011: a cross-sectional study in Minamisoma City, Fukushima prefecture

**DOI:** 10.1186/s12891-015-0711-2

**Published:** 2015-09-10

**Authors:** Shoji Yabuki, Kazuo Ouchi, Shin-ichi Kikuchi, Shin-ichi Konno

**Affiliations:** Rehabilitation Center, Fukushima Medical University Hospital, 1 Hikarigaoka, Fukushima City, Fukushima, 960-1295 Japan; Department of Orthopaedic Surgery, Fukushima Medical University School of Medicine, 1 Hikarigaoka, Fukushima City, Fukushima, 960-1295 Japan

## Abstract

**Background:**

The aim of this study was to clarify pain, quality of life and activity in the aged evacuees living in temporary housing after the Great East Japan Earthquake on 11 March 2011.

**Methods:**

The study was a cross-sectional study performed in Minamisoma City, Fukushima Prefecture 1 year and 6 months after the disaster. Inclusion criteria were the ability to walk independently and consent to answer questionnaires. Seventy-one evacuees who met the inclusion criteria were included in this study. There were 16 men and 55 women with a mean age of 75.9 years. Sixty evacuees were surveyed when they gathered at the assembly hall in the temporary housing (Assembled group) and 11 evacuees were surveyed through individual visits to their residences (Individual group). Evacuees in the Individual group agreed to participate in this study, but refused to visit the assembly hall to engage in exercise and recreation. Pain, quality of life (QOL) and level of activity were assessed with the Numeric Rating Scale (NRS), the MOS Short-Form 36 item Health Survey (SF-36) and a pedometer, respectively. Student’s t-test, Mann–Whitney U test, and Fisher’s exact test were used for statistical analysis.

**Results:**

Forty-four (62.0 %) residents had chronic pain with a mean NRS of 2.74. Twenty-one (29.6 %) of these residents had relatively severe pain rated 5 or above on the NRS. QOL was significantly lower for the subscales of “physical functioning,” “role physical”, “general health”, “social functioning”, “role emotional” and “mental health”, when compared with the national standard values. Values were also visibly lower for “physical component summary” in the summary score. On comparing the Assembled group and the Individual group, “physical function”, “role physical”, “social functioning” and “physical component summary” were found to be significantly lower in the Individual group. The mean daily number of steps was 1,892 in the Individual group and 4,579 in the Assembled group. The Individual group thus significantly took less mean daily number of steps compared with the Assembled group.

**Conclusions:**

This study quantified the state of pain, QOL and activity of aged evacuees living in temporary housing after the Great East Japan Earthquake. The evacuees frequently had chronic pain and lower physical and mental QOL scores compared to the national standard values.

## Background

On 11 March 2011, the Great East Japan Earthquake struck mainly the Pacific coast of Miyagi, Iwate and Fukushima prefectures in northeast Japan. The earthquake and subsequent tsunami caused extensive damage which resulted in radiation leakage at the Fukushima Daiichi Nuclear Power Plant. Residents living within 20 km of the power plant were consequently forced to evacuate. Since then, the residents have lived with constant anxiety of radiation exposure and its long-term consequences and impact on their lifestyles. The evacuees are scattered in and outside the prefectures and many are still living in temporary housing, more than four years after the disaster. Life in shelters in unfamiliar places is thought to have a negative impact on the physical and mental health of evacuees. The Fukushima Health Management Survey [1] clarified some of these issues. The prevalence of atrial fibrillation [2], diabetes [3], and polycythemia [4] increased in Fukushima after the Great East Japan Earthquake. Also, Yasumura reported that evacuation affected the mortality of institutionalized elderly [5]. Yabe, et al. reported that the earthquake and tsunami followed by the nuclear accident caused psychological distress among residents in Fukushima prefecture [6]. These studies suggested that the evacuees, especially elderly living in temporary housing, might have physical and mental health problems. Further, we suspected that the evacuees who gathered regularly at the assembly hall of the temporary housing facility may have had better mental health than the evacuees who did not attend these meetings. The aim of this study was to elucidate the state of the evacuees still living in temporary housing 18 months after the Great East Japan Earthquake, including both those who gathered at the assembly hall and those who did not.

## Subjects and methods

This cross-sectional study was conducted in September 2012, 18 months after the disaster.

Subjects comprised evacuees still living in temporary housing in Minamisoma City in Fukushima Prefecture located 25 km from the Fukushima Daiichi Nuclear Power Plant. There were 34 temporary housing units in Minamisoma City. Of the 6,883 individuals living in temporary housing, 2,259 were elderly individuals aged 65 years or older. Three temporary housing units were chosen for this study because they were located in the countryside of Minamisoma City and were the three units with the highest rate of elderly residents. Inclusion criteria were the ability to walk independently and consent to answer questionnaires.

Seventy-one evacuees who met the inclusion criteria were included in this study. There were 16 men and 55 women with a mean age of 75.9 ± 8.3 years. These individuals were evacuated either because their homes were washed away by the tsunami or because they lived within 20 km of the Fukushima Daiichi Nuclear Power Plant who were required to evacuate.

Sixty of these evacuees were surveyed when they gathered at the assembly hall in the temporary housing (Assembled group) and 11 evacuees were surveyed through individual visits (Individual group) to their residences. Eleven evacuees in the Individual group agreed to participate in this study, but refused to visit the assembly hall to engage in exercise and recreation with the others. Recruiting was done by City Office employees who were public health nurses. At first, the nurses delivered leaflets announcing this study to 260 temporary houses in which 356 aged evacuees were living. Enrollment by phone was accepted at the City Office. Twenty applicants at each of three housing units were included in this study on a first-come basis (Assembled group). Secondly, the nurses recruited residents who met the inclusion criteria but did not participate in the Assembly Hall meetings by visiting 23 houses and enrolling an additional 11 aged evacuees (Individual group).

This study was approved by the ethics committee of Fukushima Medical University (No. 1461) and written, informed consent was obtained from all evacuees who participated in the study.

Categories assessed were pain, quality of life (QOL) and activity. Pain was assessed with the Numeric Rating Scale (NRS) [7], QOL with the MOS Short-Form 36 item Health Survey (SF-36) [8, 9], and the activity was quantified with a pedometer (Health Counter® HJ-720IT, OMRON Healthcare Co., Ltd., Japan) which was worn for one month.

For statistical examination, Student’s t-test was used for comparing subscales of SF-36 between the values of the evacuees and the national standard values. The Mann–Whitney U test was used for comparing daily number of steps and subscales of SF-36 between the Assembled group and the Individual group. Fisher’s exact test was used for comparing the characteristics between the Assembled group and the Individual group. A *p*-value of less than 5 % was considered to indicate a significant difference. All of *p*-values in the current study were two-sided.

## Results

The characteristics of subjects are shown in Table 1. There were no significant differences between the Assembled group and the Individual group.

**Table 1:** Characteristics of subjects

	Assembled group (*n* = 60)	Individual group (*n* = 11)
Male/Female	12/48	4/7
Age (average ± SD)	74.8 ± 8.4	82.1 ± 4.7
Living		
Alone	12 (20 %)	4 (36 %)
With husband/wife	33 (55 %)	5 (45 %)
With son/daughter	15 (25 %)	2 (19 %)
Comorbidities		
Hypertension	19 (32 %)	6 (55 %)
Diabetes mellitus	7 (12 %)	1 (17 %)
Heart disease	2 (3 %)	0 (0 %)
Hyperlipemia	10 (17 %)	1 (17 %)
Brain infarction	5 (8 %)	0 (0 %)
Others	10 (17 %)	4 (36 %)

There were no significant differences between the Assembled group and the Individual group

### Pain

**Fig. 1:**
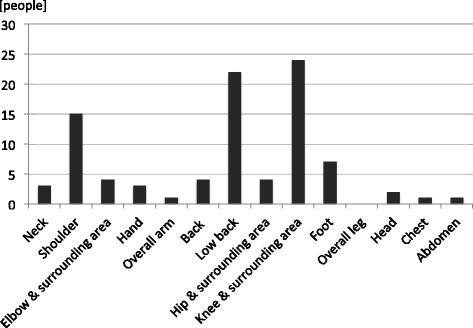
Sites of pain and number of people with pain (multiple answers allowed). Pain was common in the knee and surrounding area, and the low back

Forty-four (62.0 %) residents had chronic pain with a mean NRS score of 2.74. Twenty-one (29.6 %) of these residents had relatively severe pain rated five or above on the NRS. The site of pain was most commonly the knee and surrounding area, as well as the low back (Fig. 1).

### QOL

**Fig. 2:**
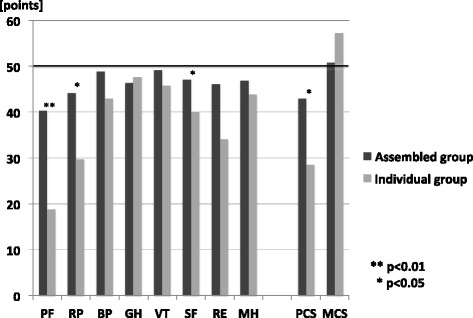
SF-36 comparing between the Assembled group and the Individual group. The Individual group had statistically significantly lower QOL than the Assembled group. Asterisks indicate significant differences between the Assembled group and the Individual group (**p < 0.01, *p < 0.05). PF: physical functioning, RP: role physical, BP: bodily pain, GH: general health, VT: vitality, SF: social functioning, RE: role emotional, MH: mental health, PCS: physical component summary, MCS: mental component summary

When compared with the national standard values [4, 5], QOL was significantly lower for the subscales of “physical functioning,” “role physical “, “general health”, “social functioning”, “role emotional” and “mental health.” Values were also visibly lower for “physical component summary” in the summary score (Table 2). On comparing the Assembled group and the Individual group, values were found to be significantly lower in the Individual group for the subscales of “physical function,” “role physical” and “social functioning.” Moreover, the Individual group had visibly lower values for “physical component summary” in the summary score (Table 2 and Fig. 2).

**Table 2:** SF-36

Group	PF	RP	BP	GH	VT	SF	RE	MH	PCS	MCS
Total	36.9 ± 17.6*	41.8 ± 15.6*	47.8 ± 11.8	46.5 ± 10.3*	48.5 ± 9.6	45.9 ± 11.9*	44.1 ± 14.8*	46.3 ± 10.8*	40.6 ± 15.9*	51.7 ± 10.4
Assembled	40.2 ± 15.0	44.1 ± 13.8	48.8 ± 11.3	46.3 ± 10.8	49.1 ± 9.2	47.0 ± 12.0	46.0 ± 13.2	46.8 ± 10.8	42.9 ± 14.5	50.7 ± 10.3
Individual	18.8 ± 20.0**	29.7 ± 19.3***	42.9 ± 13.6	47.6 ± 7.0	45.7 ± 11.4	40.0 ± 9.7***	34.1 ± 19.3	43.8 ± 10.7	28.5 ± 17.9***	57.1 ± 10.1

Data were shown as mean value ± standard deviation

*PF* physical functioning, *RP* role physical, *BP* bodily pain, *GH* general health, *VT* vitality, *SF* social functioning, *RE* role emotional, *MH* mental health, *PCS* physical component summary, *MCS* mental component summary

In Total, * indicates significant difference comparing with the national standard values, 50 (*p* < 0.01). In the Individual group, ** indicates significant difference comparing with the Assembly group (*p* < 0.01), and *** indicates significant difference comparing with the Assembly group (*p* < 0.05)

### Activity

When compared with the mean number of steps of those aged 70 years or older listed in the outline of the Ministry of Health, Labour and Welfare’s National Health and Nutrition Survey results [10], activity of overall evacuees was above in 32 % (23 people) and below average in 59 % (42 people). Data of six people (9 %) could not be obtained because they did not completely put pedometers on the bodies. On examining the Assembled group and the Individual group separately, 37 % of residents showed a larger number of steps in the Assembled group. However, only 9 % of residents in the Individual group took a larger number of steps. The mean daily number of steps was 1,892 in the Individual group and 4,579 in the Assembled group. When compared as mean daily number of steps, the Individual group took significantly less number of steps compared with the Assembled group (*p* = 0.003).

## Discussion

This study revealed that evacuees living in temporary housing had decreased QOL and a low level of activity. Moreover, 29.6 % had relatively severe chronic pain. These trends were particularly noticeable in evacuees who did not gather at the assembly hall.

### Pain

Epidemiological studies on pain in Japan have reported chronic pain at a frequency of 13 % to 22 % [11–14]. In this study, 29.6 % of evacuees had chronic pain, suggesting that many evacuees suffered from chronic pain measured 18 months after the evacuation. Elderly individuals often have pain associated with degeneration and deformation of the musculoskeletal system. However, the results of this study revealed that many were on the verge of developing disuse syndrome, which may have been accompanied by pain associated with immobility [15] and pain caused by psychogenic factors such as anxiety and depression [16–18]. In other words, the chronic pain of residents in temporary housing can be characterized as a bio-psycho-social pain syndromes [19]. Respective interventions such as orthopedic treatment, rehabilitation and psychiatric treatment may therefore be appropriate.

### QOL

The Fukushima Health Management Survey [1] showed that the prevalence of atrial fibrillation [2], diabetes [3], and polycythemia [4] increased in Fukushima after the Great East Japan Earthquake. Also, the disaster caused psychological distress among residents in Fukushima prefecture [6]. Thus, the disaster caused not only physical but also mental health problems.

The current study was performed on the evacuees living in temporary housing. Nagata, et al. reported follow-up study of the general physical and mental health of people living in temporary housing at 10 (time 1) and 20 months (time 2) after the Great East Japan Earthquake [20]. They showed that 31.0 % at time 1 and 39.0 % at time 2 were “fair” or “poor” in general health status, and 37.5 % at time 1 and 43.5 % at time 2 were at risk for psychological distress. This study was performed in Iwate prefecture located about 190 km from the Fukushima Daiichi Nuclear Power Plant where it was thought the residents might have little anxiety of radiation exposure. Shimazaki et al. reported that the values of SF-36 were significantly lower than the Japanese standard values in almost all subparts [21]. This study was performed in Minamisoma City 1 year 5 months after the disaster. The study showed similar results to the current study, however activity was not measured objectively. It has been 28 years since the 1986 Chernobyl nuclear accident. Adams et al. revealed in a report on the health of Chernobyl residents after the nuclear accident that the evacuees had a low level of mental health even 19 years after the accident [22]. Meanwhile, Bromet et al. reported that many mothers with young children suffered from depression, anxiety and post-traumatic stress disorder even though 25 years had passed since the Chernobyl nuclear accident [23]. Beehler et al. reported that almost 20 years after the Chernobyl nuclear accident, the level of mental health was more associated with chronic daily stressors than radioactive contamination [24]. Although the concentration of radiation in the region where people currently live in Fukushima is by no means high [25], residents live with anxiety over the effects of low-dose radiation. Thus, it seems useful to focus on the scientific understanding and public monitoring of the consequences to long-term, low-dose radiation.

### Activity

Ohira, et al. reported that an increase in the number of people with lifestyle-related diseases was seen in relation to the increase of body weight and obesity, but this trend appeared stronger in the evacuees since this was likely to occur due to changes in lifestyle from before to after the earthquake [26]. The increase of body weight and obesity might be attributed in part to decrease of activity. However, activity was not measured. With respect to the degree of activity exhibited by the evacuees in our study, it should be noted that those who did not gather at the assembly hall had a lower QOL and level of activity than those who did. Only individuals who were able to walk independently were surveyed in this study, and those that did not visit the assembly hall did so presumably because they could not walk. However, some individuals may not have participated because they did not feel like going out of their residences. Considering their low usual level of activity in daily life, these individuals may not have avoided just the invitation to participate this time, but may have also avoided participating in other meetings. Thus, we suggest that an environment needs to be created that encourages individuals to increase the frequency of outings, as this may increase their level of activity and QOL.

### Limitation

This study had several limitations. The first was the small subject sample. This study looked at 71 subjects, which is only 3.1 % of all aged evacuees. The second limitation was that subjects comprised only elderly individuals. A study including mothers with young children for example, may have produced different results [27]. The third limitation was that only individuals who could walk independently were included. It is expected that individuals with declining physical function may have poorer QOL, and this was implicated in our data. The fourth limitation was that this was a cross-sectional study conducted 1 year 6 months after the disaster. There were no data before the disaster. Changes in the health of evacuees will need to be tracked in a longitudinal study.

This study revealed the state of pain, QOL and activity of evacuees. On-going orthopaedic, rehabilitation and psychiatric interventions quantified by the tools have used here will hopefully result in increased activity and QOL in these people while also reducing their levels of pain.

## Conclusion

The state of pain, QOL and activity of the aged evacuees still living in temporary housing in Fukushima 18 months after the Great East Japan Earthquake was revealed. The evacuees frequently have chronic pain and have lower physical and mental QOL compared to the national standard value. Activity was also frequently low and tended to be marked in those evacuees who did not gather at assembly halls.

## Abbreviations

NRS: Numerical rating scale
QOL: Quality of life
SF-36: MOS short-form 36 item health survey
